# Mathematical Description of the RAFT Copolymerization of Styrene and Glycidyl Methacrylate Using the Terminal Model

**DOI:** 10.3390/polym14071448

**Published:** 2022-04-01

**Authors:** José Alfredo Tenorio-López, Juan José Benvenuta-Tapia, Norma García-Navarro, Eduardo Vivaldo-Lima, Pascale Champagne, Enrique Saldívar-Guerra

**Affiliations:** 1 Facultad de Ciencias Químicas, Universidad Veracruzana (UV), Coatzacoalcos 96535, Mexico; altenorio@uv.mx (J.A.T.-L.); nogarcia@uv.mx (N.G.-N.); 2 Facultad de Química, Departamento de Ingeniería Química, Universidad Nacional Autónoma de México, Ciudad de México 04510, Mexico; 3 Faculty of Engineering and Applied Science, Queen’s University, Kingston, ON K7L3N6, Canada; pascale.champagne@queensu.ca; 4 Polymer Synthesis Department, Centro de Investigación en Química Aplicada (CIQA), Saltillo 25294, Mexico

**Keywords:** glycidyl methacrylate, styrene, kinetic modeling, RAFT copolymerization

## Abstract

A mathematical model for the kinetics, composition and molar mass development of the bulk reversible addition-fragmentation chain transfer (RAFT) copolymerization of glycidyl methacrylate (GMA) and styrene (St), at several GMA molar feed fractions at 103 °C, in the presence of 2-cyano isopropyl dodecyl trithiocarbonate as the RAFT agent and 1,1′-azobis(cyclohexane carbonitrile), as the initiator, is presented. The copolymerization proceeded in a controlled manner and dispersities of the copolymers remained narrow even at high conversions. Experimental data and calculated profiles of conversion versus time, composition versus conversion and molar mass development for the RAFT copolymerization of St and GMA agreed well for all conditions tested, including high-conversion regions. The kinetic rate constants associated with the RAFT- related reactions and diffusion-controlled parameters were properly estimated using a weighted nonlinear multivariable regression procedure. The mathematical model developed in this study may be used as an aid in the design and upscaling of industrial RAFT polymerization processes.

## 1. Introduction

Functionalized copolymers are relevant at the industrial level due to their participation in chain extension, crosslinking, and polymer grafting reactions. The applications of these copolymers are diverse. They can be used as dispersants, surfactants, surface modifiers, compatibilizers, and drug delivery matrices [1]. Poly(styrene-co-glycidyl methacrylate) (St-GMA copolymers) are very interesting functional polymers. The epoxide group is useful for the chemical modification of copolymers, leading to a wide variety of applications. The glycidyl functional group has potential applications in functionalization with amines [1], compatibilized polymer blends [2], acids [3], cation-exchange adsorbents [4], and the chain extension of polyesters [5,6], leading to the production of materials with increased melt viscosities and strengths.

Industrial production of St-GMA copolymers is usually carried out by conventional free-radical polymerization (FRP) processes [7,8]. FRP has the advantages of undemanding operation conditions and versatility of monomers that can be used. The negative aspects of FRP include poor control of the molar mass and polymer microstructure of the product. St-GMA copolymers synthesized by FRP typically possess broad molar mass distributions [9].

Another disadvantage of FRP processes is temperature control. For instance, the free radical copolymerization of St and GMA proceeds very rapidly and exothermically. Therefore, efficient heat removal is required to avoid reaching reactor-runaway conditions, which may result in the production of out-of-specification materials.

Reversible-deactivation radical polymerization (RDRP), also known as controlled radical polymerization (CRP), is important because it allows the synthesis of copolymers with not only narrow molar mass distributions and well-defined microstructures, but also more homogeneous composition distributions, compared to FRP [10].

The main RDRP techniques, namely, nitroxide-mediated polymerization (NMP) [11,12], atom transfer radical polymerization (ATRP) [13], and reversible addition-fragmentation chain transfer polymerization [14], have allowed the synthesis of functional polymers with predefined molar masses, low dispersity values, and defined microstructures.

Recently, the incorporation to RAFT polymerization of photoinduced electron/energy transfer (PET) and polymerization-induced self-assembly (PISA) methodologies into RAFT polymerization has been applied to the polymerization of various functional monomers, resulting in good control of molecular weight and molecular weight distributions, similar to thermally initiated systems, with supplementary advantages, such as mild reaction conditions and low energy consumption [15,16]. PET-RAFT polymerization was successfully conducted, obtaining polymer products with controlled molecular weights and narrow dispersities (Đ = 1.02–1.13) [15], while RAFT-PISA was successfully applied to the synthesis of block copolymer nano-objects with different morphologies [16].

Few experimental reports on the RAFT copolymerization of St and GMA are available, and to the best of our knowledge there are no reported studies on the production of functional St-GMA copolymers synthesized by RAFT polymerization above 100 °C [17], which is the range of interest in the synthesis of acrylate-containing copolymers.

As far as we are aware, only the syntheses of St-GMA copolymers by atom transfer radical polymerization using copper-based catalyst systems in bulk and in toluene, at 60 °C [18], and by nitroxide mediated polymerization in 50 wt.% 1,4-dioxane solution, at 90 °C, have been reported [19].

The control of polymer microstructural parameters, such as copolymer composition, copolymer sequence distributions and molar mass dispersities, is important in copolymerization processes. Parameter estimation of kinetic rate constants associated with copolymerization processes is important for accurate calculation of polymerization rates and copolymer sequence distributions, which are fundamental to the production of copolymers with predefined properties.

The application of fundamental polymerization models, including kinetic and reactor models, is necessary to understand the mechanisms and phenomena behind these processes. Kinetic studies are particularly important because they provide better control strategies during the production of polymers at an industrial scale [20]. These models can be used to calculate monomer conversion, polymer microstructure, molar mass averages, and full molar mass distributions (MMD) under various operating conditions. Furthermore, mathematical modeling may be used to better understand and operate polymerization processes, allowing the prediction of the effect of operating conditions on polymerization rate and polymer properties; a good model may simplify experimental programs. The industrial production of polymer materials usually involves high-temperature processes. Therefore, it is important to carry out kinetic studies at similarly high temperatures. To the best of our knowledge, the kinetic modeling of the RAFT copolymerization of St and GMA has not been reported so far. It is known that RAFT agents alter the concentration profiles of active species, compared to FRP [20,21,22]. This situation may favor the preferential incorporation of one of the comonomers, affecting both, copolymer composition and polymerization kinetics [21].

In this contribution, the kinetic modeling of the RAFT bulk copolymerization of St and GMA using 2-cyano-2-propyl dodecyl trithiocarbonate (CPDT) as a RAFT agent, and 1,1′-azobis (cyclohexane carbonitrile) as an initiator, at different levels of GMA content, is reported. Our objectives were to estimate the rate coefficients for RAFT reactions involved in the polymerization scheme and evaluate the performance of the model by comparing model predictions against experimental data generated in our laboratory. In developing the model, it was assumed that the terminal model is valid and that the RAFT activation and transfer cycles proceed with the same kinetic parameters. Diffusion-controlled termination was considered using a model based on free-volume theory.

## 2. Experimental

### 2.1. Reagents

GMA (97%) and styrene (99%) were purchased from Aldrich (Saint Louis, MO, USA) and purified as explained in one of our earlier studies. [9] 1,1′-azobis(cyclohexane carbonitrile) (ACHN, 98%) and 2-cyano-2-propyl dodecyl trithiocarbonate (97%) were both purchased from Aldrich (Saint Louis, MO, USA) and used as received.

### 2.2. Size Exclusion Chromatography (SEC) Characterization Method

Number- and weight-average molar masses (M_n_ and M_w_, respectively), and molar mass dispersity (Ð = M_w_/M_n_) of the synthesized polymers were measured using a Waters 1515 gel permeation chromatograph (GPC, Waters, Milford, MA, USA) equipped with a refractive-index detector as well as HR 1, HR 3, and HR 4 columns. Calibration procedure and operating conditions were the same as described in an earlier report from our group [19].

### 2.3. ^1^H-NMR Characterization Method

^1^H-nuclear magnetic resonance (^1^H-NMR) spectra were obtained using a Varian 300 MHz spectrometer (Varian, Santa Clara, CA, USA) using deuterated chloroform (CDCl_3_) as a solvent and tetramethylsilane (TMS) as an internal standard, at room temperature. Sample preparation and measurement proceeded as reported in our previous studies. [14] Data analysis for determination of copolymer composition and sequence distribution of monomer units from ^1^H-NMR characteristic signals was carried out using the appropriate equations [23].

### 2.4. Copolymerization Reactions

Bulk copolymerizations of St and GMA, at 103 °C, proceeded in a 1-L high-pressure stainless-steel jacketed reactor (Parr Model 4523, Moline, IL, USA) with temperature control, as well as pressure and stirring sensors. Ultra-high-purity nitrogen was used to provide an inert environment. Appropriate amounts of GMA and St were then added to the reactor, followed by the initiator and RAFT agent. A mixing rate of 150 rpm was used. The polymerizations proceeded under nitrogen atmosphere, at 4.14 bar. Then, temperature was increased to 103 °C using a Huber Unistat 815w thermoregulator, in cascade mode. The thermal oil flew directly to the reactor jacket and to the coil, which ensured temperature control with a precision of ±1 °C. Sample withdrawal, preparation and analyses proceeded as detailed in one of our earlier studies [19].

Monomer conversion was not measured directly. We measured polymer yield, which was calculated gravimetrically, as the ratio of mass of produced polymer to mass of initial total monomer. Therefore, although we refer to monomer conversion in the figures of this contribution, when referring to experimental data, it is strictly polymer yield.

## 3. Model Development

A kinetic mathematical model was developed to calculate polymerization rate, evolution of molar mass averages, and copolymer composition. The model contains the following assumptions: (a) penultimate effects were neglected; (b) while in some reactions the intermediate macroradical species formed during the additional step of the RAFT process may be stable enough to delay polymerization (maximum lifetime of 1 s), it is not considered here to initiate new species and terminate [24,25,26,27]; and (c) branching has also been neglected.

The starting polymerization scheme is shown in Table 1. Three polymer populations are involved: propagating radical or active (living) polymer molecules with terminal units A or B (P_n_ and Q_n_), dormant polymer molecules with terminal units A or B (TP_n_ and TQ_n_), and dead polymer molecules (*M*_n_), where subscript *n* is the number of monomeric units in the macromolecule. A and B represent St and GMA terminal units, respectively. T is the RAFT agent.

The mathematical model developed in this contribution is based on the method of moments. The definitions of moments of several polymer species are shown in Table 2.

The detailed kinetic equations for low molar mass and polymer species are summarized in Table 3.

Table 4 shows the obtained moment equations.

Overall monomer conversion, copolymer composition and average molar masses, M_n_ and M_w_ are calculated using Equations (26)–(32). M in Equations (26) and (27) stands for monomer content; subscripts 1, 2 and 0 stand for monomer 1, monomer 2, and initial conditions, respectively.

(26) Overall conversion = (M_1o_ + M_2o_ − (M_1_ + M_2_))/(M_1o_ + M_2o_)

(27) Copolymer composition: F_1_ = (M_1o_ − M_1_)/((M_1o_ − M_1_) + (M_2o_ − M_2_))

(28) $$\mathrm{Number}\text{-}\mathrm{average}\;\mathrm{chain}\;\mathrm{length}:\;r_{N}=\frac{Y_{1}^{a}+{\;Y}_{1}^{b}+{\;Z}_{1}^{a}+{\;Z}_{1}^{b}+{\;D}_{1}}{Y_{0}^{a}+{\;Y}_{0}^{b}+{\;Z}_{0}^{a}+{\;Z}_{0}^{b}+{\;D}_{0}}$$

(29) $$\mathrm{Weight}\text{-}\mathrm{average}\;\mathrm{chain}\;\mathrm{length}:\;r_{w}=\frac{Y_{2}^{a}+{\;Y}_{2}^{b}+{\;Z}_{2}^{a}+{\;Z}_{2}^{b}+{\;D}_{2}}{Y_{1}^{a}+{\;Y}_{1}^{b}+{\;Z}_{1}^{a}+{\;Z}_{1}^{b}+{\;D}_{1}}$$

(30) $$\mathrm{Dispersity}:\;Ð=\frac{r_{w}}{r_{N}}$$

(31) $$M_{n}=r_{N}\;(F_{1}\;{\mathrm{PM}}_{1}+F_{2}\;{\mathrm{PM}}_{2})$$

(32) M_w_ = Ð M_n_

The kinetic rate constants and parameters required by the model are summarized in Table 5. The values of the reactivity ratios for the RAFT copolymerization of St and GMA were obtained using a weighted non-linear multivariable regression approach, using software RREVM [19]. These values are also provided in Table 5.

The mobility of high-molar-mass macromolecules is reduced at high conversions in FRP. Consequently, the rates of termination, propagation and RAFT reactions involving polymer molecules change throughout the reaction. In this study, diffusion-controlled (DC) effects were considered only for the termination reactions (auto-acceleration (AA) effect), using Equations (33) and (34), where k_t_ is an effective kinetic rate constant, $k_{t}^{o}$ is the corresponding intrinsic kinetic rate constant, V_fo_ and V_f_ are the initial and final free-volume fractions, respectively, and ${\beta k}_{\mathrm{kt}}$ is a free-volume parameter. It was assumed that ${\beta k}_{\mathrm{tc}}$ = ${\beta k}_{\mathrm{td}}$, parameters to be evaluated as AA effect.

(33) $$k_{\mathrm{tc}}=k_{\mathrm{tc}}^{o}\;\exp\left[-{\beta k}_{\mathrm{tc}}\left(\frac{1}{V_{f}}-\frac{1}{V_{\mathrm{fo}}}\right)\right]$$

(34) $$k_{\mathrm{td}}=k_{\mathrm{td}}^{o}\;\exp\left[-{\beta k}_{\mathrm{td}}\left(\frac{1}{V_{f}}-\frac{1}{V_{\mathrm{fo}}}\right)\right]$$

The free-volume fraction, V_f_, is calculated using Equation (35) [33].

(35) $$V_{f}=[0.025+\alpha_{p}(T-T_{g,p}\;)]\;\varphi_{p}+[0.025+\alpha_{M_{1}}(T-T_{g,M_{1}})]\;\varphi M_{1}+[0.025+\alpha_{M_{2}}(T-T_{g,M_{2}})]\;\varphi M_{2}$$

α in Equation (35) is the thermal expansion coefficient, φ is the volume fraction, and T_g_ is the glass transition temperature. Subscripts p and M_i_ denote polymer and monomer i, respectively. T_gp_ is estimated using the Fox expression, given by Equation (36) [34].

(36) $$T_{g,p}=1/{\left[\frac{f_{P1}}{T_{g,P_{1}}{}_{}}+\frac{f_{P2}}{T_{g,P_{2}}{}_{}}\;\right]}_{\;}$$

f_p_ in Equation (36) is the weight fraction of the polymer. Table 6 shows the physical properties of monomers and polymers used.

Although there are many mathematical models for DC effects in FRP and step-growth polymerization processes available in the literature, it is difficult to adequately describe the performance of different monomers under wide ranges of operating conditions using a single model with a single set of parameters. One such model is the Marten-Hamielec (MH) model [43,44], but it has the disadvantage of being discontinuous and requires an onset trigger criterion. Attempts to remove the trigger criterion resulted in a simpler, but less accurate model [45]. Therefore, in this study, we used the simplified version of the MH model [45], with a simpler onset trigger criterion, which causes it to be closer to the original model.

The assumptions summarized in Table 7 allow the determination the kinetic rate constants of RAFT activation and transfer for homopolymerizations of St and GMA. The assumptions indicated in the columns of Table 7 (e.g., k_aa1_ = k_at1_) are necessary, due to the absence of experimental data to isolate the contributions of the two RAFT cycles to the properties of the produced polymer. Some authors have argued that the kinetic constants of the RAFT activation and RAFT transfer cycles may be different [46,47,48]; in RAFT polymerization modeling work, the equality of the kinetic constants of the RAFT cycles is supported [49,50,51,52].

Another assumption is that the kinetic rate constants associated with the dormant species [TP_n_] and [TQ_n_] (k_at3_, k_ft3_, k_at4_, k_ft4_) can be approximated from the Mayo-Lewis terminal model [53]. This is achieved by considering the four reactions present in the RAFT transfer cycle and performing only consumption balances for the [TP_n_] and [TQ_n_] species, which are complemented with consumption balances for the [TP_r_] and [TQ_r_] species to complete the cycle. By calculating this, Equations (37)–(42) were obtained.

(37) $$\frac{d\;\left[{\mathrm{TP}}_{n}\right]}{d\;\left[{\mathrm{TQ}}_{n}\right]}=\frac{k_{\mathrm{ft}3}\left[{\mathrm{TP}}_{n}\right]\left(r_{3}\left[P_{r}\right]+\left[Q_{r}\right]\right)}{k_{\mathrm{ft}4}\left[{\mathrm{TQ}}_{n}\right]\left(r_{4}\left[Q_{r}\right]+\left[P_{r}\right]\right)}$$

where:

(38) $$r_{3}=\frac{k_{\mathrm{ft}1}}{k_{\mathrm{ft}3}}$$

(39) $$r_{4}=\frac{k_{\mathrm{ft}2}}{k_{\mathrm{ft}4}}$$

(40) $$\frac{d\;\left[{\mathrm{TP}}_{r}\right]}{d\;\left[{\mathrm{TQ}}_{r}\right]}=\frac{k_{\mathrm{at}4}\left[{\mathrm{TP}}_{r}\right]\left(r_{5}\left[P_{n}\right]+\left[Q_{n}\right]\right)}{k_{\mathrm{at}3}\left[{\mathrm{TQ}}_{r}\right]\left(r_{6}\left[Q_{n}\right]+\left[P_{n}\right]\right)}$$

where:

(41) $$r_{5}=\frac{k_{\mathrm{at}1}}{k_{\mathrm{at}4}}$$

(42) $$r_{6}=\frac{k_{\mathrm{at}2}}{k_{\mathrm{at}3}}$$

Additionally, $r_{1}$ = $r_{3}$ = $r_{5}$ and $r_{2}$ = $r_{4}$ = $r_{6}$. This is due to the application of the terminal model to the RAFT-transfer cycle. The RAFT-activation and -transfer kinetic parameters corresponding to homopolymerization of styrene and GMA were estimated using homopolymerization data for each monomer.

RAFT-related kinetic rate constants (optimization A) and AA effect parameters (optimization B) were estimated from overall conversion (X)-time and M_n_-time experimental results, using a weighted non-linear multivariable regression procedure where the residual variance was minimized. The objective function is defined in Equation (43).

(43) $$\mathrm{Objective}\;\mathrm{function}=\min\;\left[\sum_{i}^{n}\frac{1}{\sigma_{X}^{2}}{\left(X_{i}^{e}-{\;X}_{i}^{c}\right)}^{2}+\sum_{i}^{n}\frac{1}{\sigma_{M}^{2}}{\left(M_{i}^{e}-{\;M}_{i}^{c}\right)}^{2}\right]$$

**Figure 1:**
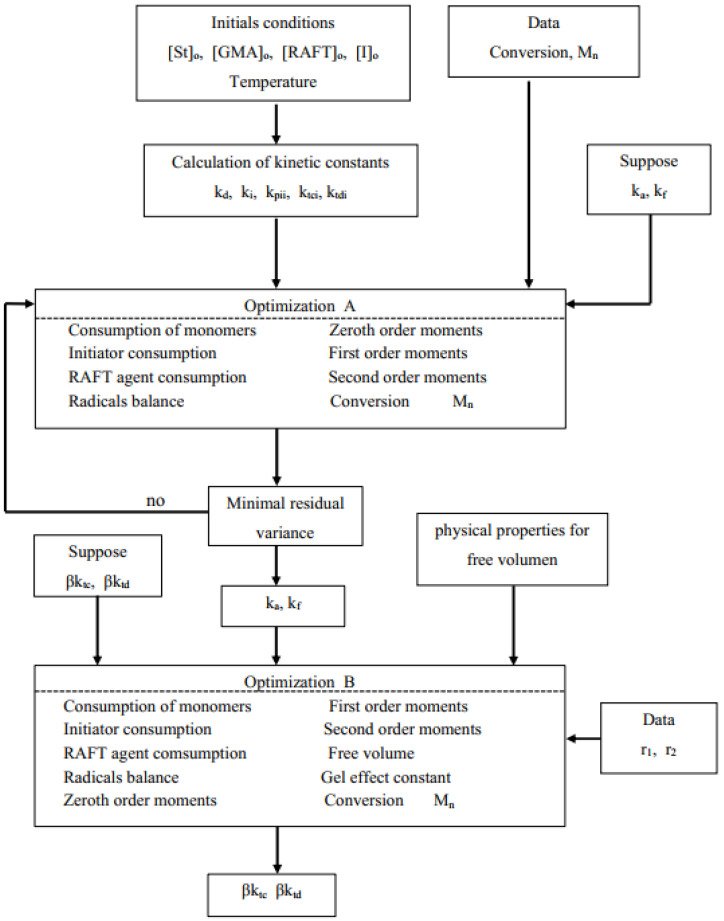
Flow chart for the estimation of kinetic and AA effect parameters.

Superscripts e and c in Equation (43) stand for experimental and calculated values, respectively; σ_X_^2^ and σ_M_^2^ are variances of conversion and molar mass data, respectively; and n is the number of data points in each experimental data set. However, for simplicity, both variances were assumed equal to one. Optimization A was carried out using St and GMA homopolymerization data only, using the model without the AA terms. Optimization B was conducted for each copolymerization data set. The parameters obtained from each data set were regressed to obtain the final estimates. The flow chart that describes the modeling and parameter estimation strategies used in this contribution is shown in Figure 1. The model equations were solved using an in-house Fortran code. The optimization procedure for parameter estimation was carried out with the subroutine UWHAUS [54]. The system of ordinary differential equations was solved using subroutine DDASSL [55].

## 4. Results

RAFT Synthesis and Characterization of Reactive Copolymers

**Figure 2:**
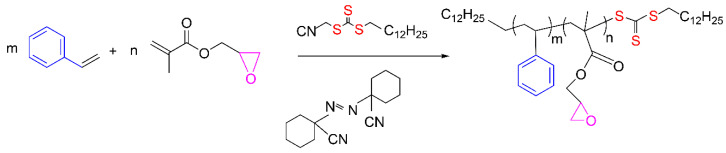
Simplified polymerization scheme.

St-GMA copolymers of different compositions (f_GMA_ = 0.10, 0.15, 0.30, and 0.40) were synthesized by RAFT bulk copolymerization of the monomers, at 103 °C, according to Figure 2. A M_n_ of ~30,000 g mol^−1^ was sought for all polymers. Final overall monomer conversions in a range of 85–90% were obtained. The experimental conditions used in this study are reported in Table 8.

**Figure 3:**
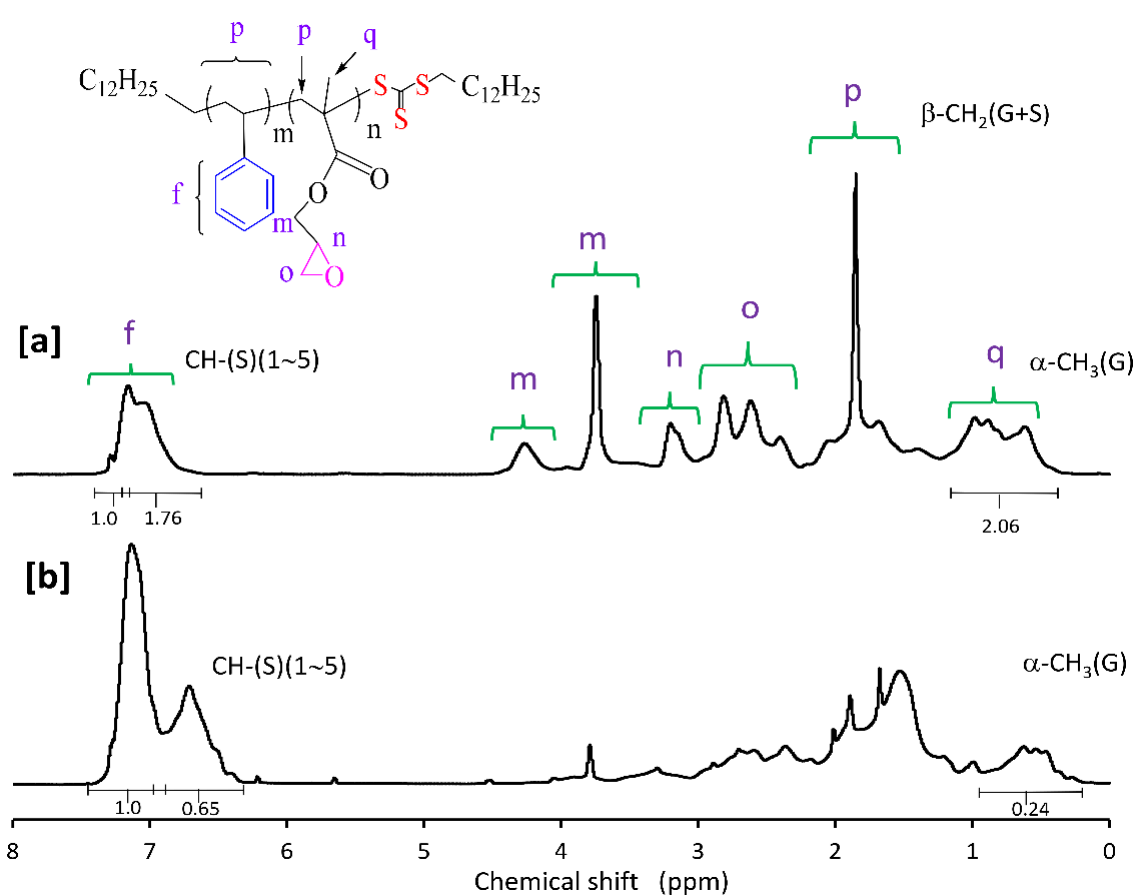
^1^H NMR spectrum of a RAFT synthesized St-GMA copolymer with (**a**) 55% and (**b**) 20% mole fractions of GMA in the feed mixture, using CPDT and ACHN. (f) phenyl protons of styrene; (m, n, o) methylene oxy (–OCH_2_–) protons of GMA; (p, q) methyl protons of GMA.

Molar compositions of the St-GMA copolymers synthesized in this study were determined from the relative areas of the ^1^H NMR characteristic signals [23,30]. ^1^H NMR spectra for some of the obtained St-GMA copolymers are shown in Figure 2. Chemical shifts from phenyl protons in the region of 6.6–7.3 ppm, and methylene oxy (–OCH_2_–) protons and methyl protons of GMA units at 3.5–4.5 and 0.5–1.2 ppm, respectively, are observed in Figure 3. The mole fraction of GMA in the copolymer was calculated as: F_2_ = 5 A_3_/(5 A_3_ + 3 A_2_), where A_2_ and A_3_ are peak areas of phenyl and methyl protons, respectively. This method was used in this work due to the distinct NMR resonance of the GMA methyl group even at low GMA mole fractions in the copolymer [30].

The St-GMA copolymers were characterized by SEC. They had M_n_ ~22,200–26,300 g mol^−1^ and Ð~1.21–1.28, which suggests that no side reactions took place and that most of the active polymer molecules remained living until the end of the polymerization.

Several kinetic models have been developed for RAFT homo- [56,57,58] and copolymerization of a few monomers [59,60,61,62]. As stated earlier, in our polymerization scheme we assumed that no branches to the adduct were produced, making it easier to model our RAFT copolymerization system using the terminal model [53], which is given by Equations (37)–(42). Therefore, the RAFT homo- and cross-propagation kinetic rate constants for the copolymerization system were determined by the corresponding values of RAFT homopolymerizations of St and GMA, and from reported values of r_1_ y r_2_ for the same copolymerization system [19].

Even though the RAFT polymerization mechanism is well-established and accepted [63,64,65,66,67], the parameters involved, such as addition, fragmentation, and termination kinetic rate constants, are not always reliable even in well-known systems, such as the RAFT homopolymerizations of methyl methacrylate and St [68]. The activation and transfer kinetic rate constants evaluated in this study for RAFT copolymerization of St and GMA are summarized in Table 9.

The profiles obtained with the parameters reported in Table 9 are not included due to space restrictions, but very good agreement is obtained in the low-conversion region, where DC effects are not observed. Although it has been reported that DC effects are important in all the reactions where polymer molecules are involved, in RAFT polymerizations [64], we considered DC termination only [69], to capture the phenomenon without adding too many additional parameters that required estimation. The AA termination parameters evaluated are provided in Table 10. As observed in Table 10 the higher the content of St in the copolymer, the higher the value of the AA termination parameter.

**Figure 4:**
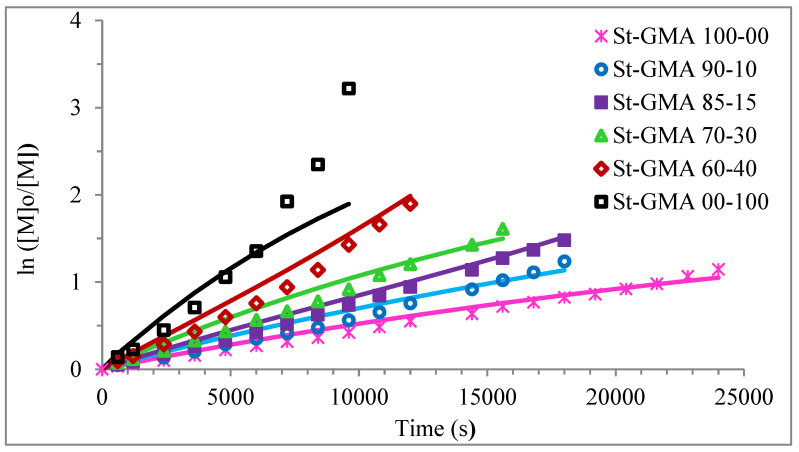
First-order of different St-GMA samples. Symbols represent experimental data, whereas solid lines correspond to model predictions.

**Figure 5:**
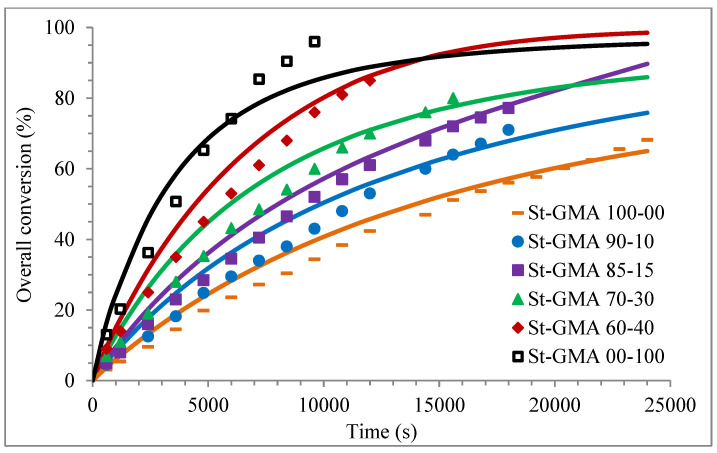
Profiles of overall conversion versus time for the different St-GMA samples. Symbols represent experimental data, whereas solid lines correspond to model predictions.

**Figure 6:**
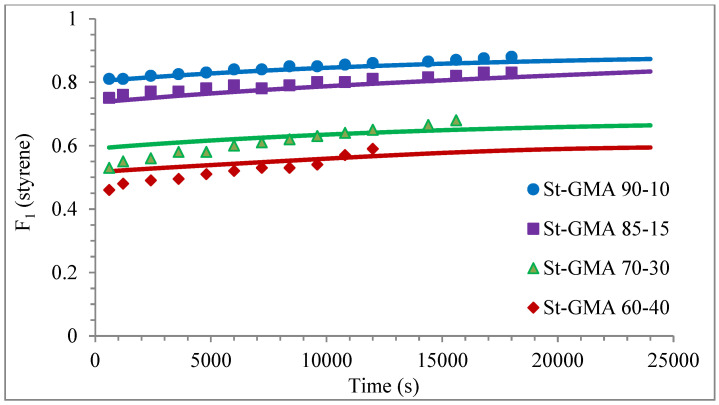
Copolymer composition versus time profiles for the different St-GMA samples. Symbols represent experimental data, whereas solid lines correspond to model predictions.

**Figure 7:**
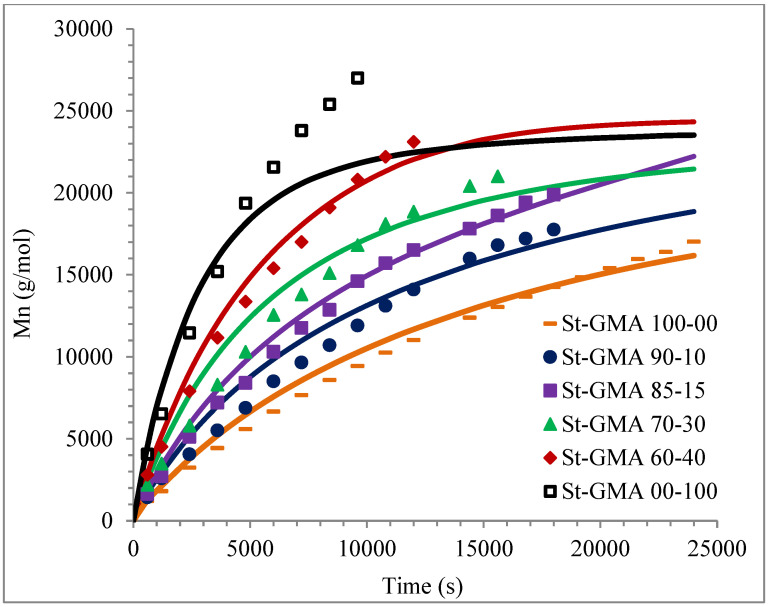
Profiles of M_n_ versus time for St-GMA samples. Symbols and solid lines correspond to experimental and calculated profiles, respectively.

**Figure 8:**
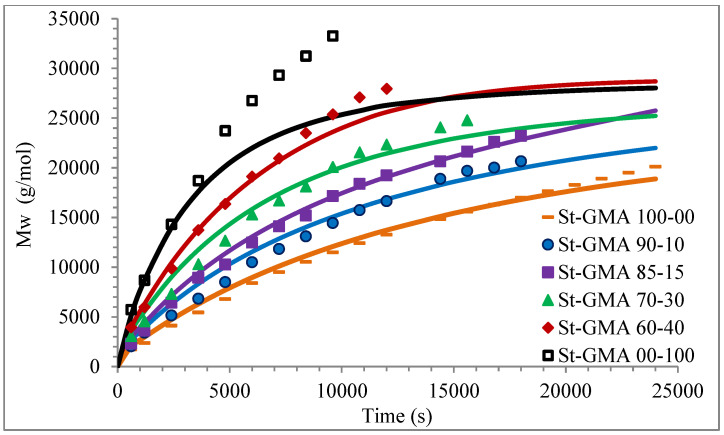
Profiles of M_w_ versus time for the different St-GMA samples. Symbols and solid lines correspond to experimental and calculated profiles, respectively.

**Figure 9:**
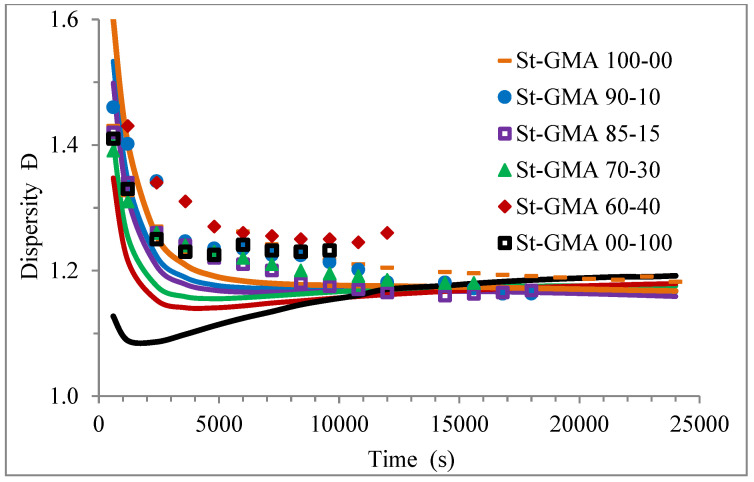
Profiles of dispersity versus time for the different St-GMA samples. Symbols and solid lines correspond to experimental and calculated profiles, respectively.

**Figure 10:**
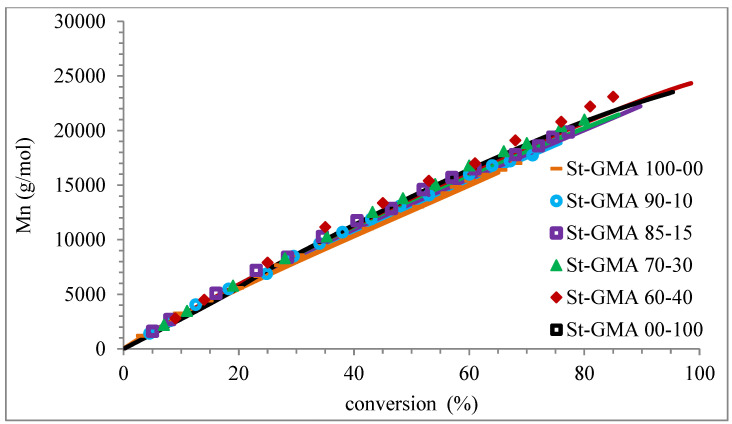
Profiles of M_n_ versus conversion for the different St-GMA samples. Symbols and solid lines correspond to experimental and calculated profiles, respectively.

Figure 4 shows a first order behavior plot. A comparison of experimental data and calculated profiles of conversion, M_n_, M_w_, and dispersity versus time (or conversion, in one case) is shown in Figure 5, Figure 6, Figure 7, Figure 8, Figure 9 and Figure 10.

The AA effect occurs at high conversions for the copolymerization reactions of St-GMA. Considering this, the determination of the kinetic rate constants associated with the RAFT cycles and the parameters associated with the termination reactions were carried out independently, which helped minimize possible correlations.

Except for sample St-GMA 00-100 where some discrepancies between experimental data and calculated profiles of conversion versus time were obtained (see Figure 5), the agreement is good in all other cases. Regarding copolymer composition, the agreement between calculated and experimental profiles of F_1_ versus conversion is good when St content is high, but some deviations are observed in low conversions when its content decreases (see Figure 6).

Calculated and experimental profiles of M_n_ and M_w_ versus time are compared in Figure 7 and Figure 8, respectively. Once again, the agreement is good except for sample S-GMA 00-100, where some discrepancies are observed. Considering the results obtained with the adjustment in high conversions, it is not enough to consider only diffusion-controlled effects on the termination kinetic rate constants for GMA homopolymerization (St-GMA 00-100).

Figure 9 shows how the dispersity of the produced copolymers evolves over time. Figure 9 and Figure 10 show that the model can describe the controlled/living behavior of this RDRP system. Figure 10 shows the typical linear behavior of an M_n_ versus conversion profile for an RDRP system.

**Figure 11:**
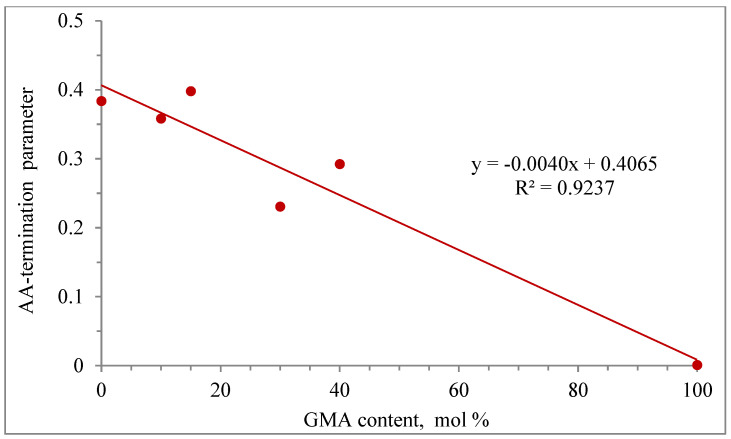
Linear regression of the AA-termination parameters vs. GMA content.

The effect of GMA content on the AA-termination parameter (βk_t_) is shown in Figure 11. A linear behavior of the AA termination parameter as a function of GMA content is observed, with an R^2^ correlation of 0.9237.

As observed from this modeling study, AA-termination parameters for each copolymerization case were needed to obtain good results, but as observed in Figure 11, a linear trend was obtained for the GMA homopolymerization case.

## 5. Conclusions

Our model for the RAFT copolymerization of St and GMA agrees very well with the experimental data generated in our laboratory and also reported in this contribution. The reactivity ratios determined for this copolymerization system were used considering that the terminal model was also fulfilled in the RAFT activation and transfer reactions. DC-termination using a simple free-volume model was sufficient to capture the effect of DC reactions in this system.

Unlike other modeling studies where neglection of the intermediate adduct results in qualitatively correct, but quantitatively inaccurate, predictions of the behavior of a RAFT polymerization system (Model 3 of [62]), the use of activation and transfer RAFT reactions resulted in our case in both qualitatively and quantitatively correct representations of the RAFT copolymerization of St and GMA. This model can be applied to other homo- and copolymerizations, and also extended to other systems, such as photo-RAFT polymerizations [15,70,71,72].

## Figures and Tables

**Table 1:** Detailed polymerization scheme used in this study.

Initiation	Propagation
I $\overset{k_{d}f}{\to }$2 $R^{•}$	
$R^{•}$+ A $\overset{k_{i1}}{\to }$P_1_ P_n_ + A $\overset{k_{p11}}{\to }$P_n+1_ Q_n_ + A $\overset{k_{p21}}{\to }$P_n+1_	
$R^{•}$+ B →ki 2Q_1_ P_n_ + B $\overset{k_{p12}}{\to }$Q_n+1_ Q_n_ + B $\overset{k_{p22}}{\to }$Q_n+1_	
RAFT activation T + $P_{n}$$\overset{k_{\mathrm{aa}1}}{\underset{k_{\mathrm{fa}1}}{⇄}}$R^•^ + TP_n_ T + Q_n_ $\overset{k_{\mathrm{aa}2}}{\underset{k_{\mathrm{fa}2}}{⇄}}$R^•^ + TQ_n_	RAFT transfer P_n_ + TP_r_ $\overset{k_{\mathrm{at}1}}{\underset{k_{\mathrm{ft}1}}{⇄}}$TP_n_ + P_r_ P_n_ + TQ_r_ $\overset{k_{\mathrm{at}3}}{\underset{k_{\mathrm{ft}3}}{⇄}}$TP_n_ + Q_r_ Q_n_ + TQ_r_ $\overset{k_{\mathrm{at}2}}{\underset{k_{\mathrm{ft}2}}{⇄}}$TQ_n_ + Q_r_ Q_n_ + TP_r_ $\overset{k_{\mathrm{at}4}}{\underset{k_{\mathrm{ft}4}}{⇄}}$TQ_n_ + P_r_
Termination	
P_n_ + P_r_ $\overset{k_{\mathrm{tc}1}}{\to }$*M*_n+r_ P_n_ + P_r_ $\overset{k_{\mathrm{td}1}}{\to }$*M*_n_ + *M*_r_	
Q_n_ + Q_r_ $\overset{k_{\mathrm{tc}2}}{\to }$*M*_n+r_ Q_n_ + Q_r_ $\overset{k_{\mathrm{td}2}}{\to }$*M*_n_ + *M*_r_	
P_n_ + Q_r_ $\overset{k_{\mathrm{tc}3}}{\to }$*M*_n+r_ P_n_ + Q_r_ $\overset{k_{\mathrm{td}3}}{\to }$*M*_n_ + *M*_r_	

**Table 2:** Definition of moments of the several polymer species.

$Y_{i}^{a}$ = $\sum_{n=0}^{\infty }n^{i}$ [P_n_] $Z_{i}^{a}$ = $\sum_{n=0}^{\infty }n^{i}$ [TP_n_] $D_{i}$ = $\sum_{n=0}^{\infty }n^{i}$ [*M*_n_]
$Y_{i}^{b}$ = $\sum_{n=0}^{\infty }n^{i}$[Q_n_] $Z_{i}^{b}$= $\sum_{n=0}^{\infty }n^{i}$[TQ_n_] i = 0, 1, 2

**Table 3:** Kinetic equation for species.

$\frac{\mathrm{dA}}{\mathrm{dt}}$ = −k_i1_[ $R^{•}$ ][A] − k_p11_[A][ $Y_{o}^{a}$ ] − k_p21_[A][ $Y_{o}^{b}$ ] St monomer	(1)
$\frac{\mathrm{dB}}{\mathrm{dt}}$ = −k_i2_[ $R^{•}$ ][B] − k_p12_[B][ $Y_{o}^{a}$ ] − k_p22_[B][ $Y_{o}^{b}$ ] GMA monomer	(2)
$\frac{\mathrm{dI}}{\mathrm{dt}}$ = −k_d_[I] initiator	(3)
$\frac{{\mathrm{dR}}^{•}{}^{}}{\mathrm{dt}}$ = 2k_d_f[I] − k_i1_[A][ **R**•] − k_i2_[B][ ${\;R}^{•}$ ] − k_fa1_[ $Z_{o}^{a}$ ][ ${\;R}^{•}$ ] + k_aa1_[ $Y_{o}^{a}$ ][T] − k_fa2_[ $Z_{o}^{b}$ ][R^*^] + k_aa2_[ $Y_{o}^{b}$ ][T]	(4)
$\frac{\mathrm{dt}}{\mathrm{dt}}$ = k_fa2_[ $R^{•}$ ][ $Z_{o}^{b}$ ] − k_aa2_[T][ $Y_{o}^{b}$ ] + k_fa1_[ $R^{•}$ ][ $Z_{o}^{a}$ ] − k_aa1_[T][ $Y_{o}^{a}$ ] RAFT agent	(5)
$\frac{{\mathrm{dP}}_{n}}{\mathrm{dt}}$ = k_i1_[ $R^{•}$ ][A] + k_fa1_[ $R^{•}$ ][TP_n_] − k_aa1_[T][P_n_] − k_at3_[ $Z_{o}^{b}$ ] [P_n_] + k_ft3_[ $Y_{o}^{b}$ ][TP_n_] + k_ft1_[ $Y_{o}^{a}$ ][TP_n_] − k_at1_[ $Z_{o}^{a}$ ][P_n_] − k_p11_[A][P_n_] − k_p12_[B][P_n_] + k_p11_[A][P_n−1_] + k_p21_[A][Q_n−1_] − k_tc3_[ $Y_{o}^{b}$ ][P_n_] −k_td3_[ $Y_{o}^{b}$ ][P_n_] − k_tc1_[ $Y_{o}^{a}$ ][P_n_] − k_td1_[ $Y_{o}^{a}$ ][P_n_]	(6)
$\frac{{\mathrm{dQ}}_{n}}{\mathrm{dt}}$ = k_i2_[ $R^{•}$ ][B] + k_fa2_[R^*^][TQ_n_] − k_aa2_[T][Q_n_] – k_at4_[ $Z_{o}^{a}$ ][Q_n_] + k_ft4_[ $Y_{o}^{a}$ ][TQ_n_] + k_ft2_[ $Y_{o}^{b}$ ][TQ_n_] − k_at2_[ $Z_{o}^{b}$ ][Q_n_] − k_p21_[A][Q_n_] − k_p22_[B][Q_n_] + k_p12_[B][P_n−1_] + k_p22_[B][Q_n−1_] − k_tc3_[ $Y_{o}^{a}$ ][Q_n_] − k_td3_[ $Y_{o}^{a}$ ][Q_n_] − k_tc2_[ $Y_{o}^{b}$ ][Q_n_] − k_td2_[ $Y_{o}^{b}$ ][Q_n_]	(7)
$\frac{{\mathrm{dTP}}_{n}}{\mathrm{dt}}$ = −k_fa1_[ $R^{•}$ ][TP_n_] + k_aa1_[T][P_n_] + k_at3_[ $Z_{o}^{b}$ ][P_n_] – k_ft3_[ $Y_{o}^{b}$ ][TP_n_] + k_at1_[ $Z_{o}^{a}$ ][P_n_] − k_ft1_[ $Y_{o}^{a}$ ][TP_n_]	(8)
$\frac{{\mathrm{dTQ}}_{n}}{\mathrm{dt}}$ = −k_fa2_[ $R^{•}$ ][TQ_n_] + k_aa2_[T][Q_n_] − k_ft2_[ $Y_{o}^{b}$ ][TQ_n_] + k_at2_[ $Z_{o}^{b}$ ][Q_n_] + k_at4_[ $Z_{o}^{a}$ ][Q_n_] − k_ft4_[ $Y_{o}^{a}$ ][TQ_n_]	(9)
$\frac{dM_{n}}{\mathrm{dt}}$ = k_tc3_ $\left(\sum_{a=0}^{n}P_{a}Q_{n-a}\right)$ + $\frac{1}{2}$ k_tc1_ $\left(\sum_{a=0}^{n}P_{a}P_{n-a}\right)$ + $\frac{1}{2}$ k_tc2_ $\left(\sum_{a=0}^{n}Q_{a}Q_{n-a}\right)$ + k_td3_[ $Y_{o}^{b}$ ][P_n_] + k_td1_[ $Y_{o}^{a}$ ][P_n_] + k_td2_[ $Y_{o}^{b}$ ][Q_n_] + k_td3_[ $Y_{o}^{a}$ ][Q_n_]	(10)

**Table 4:** Moment equations for the species present in the St-GMA copolymerization.

Zeroth-order moments	
$\frac{{\mathrm{dY}}_{o}^{a}}{\mathrm{dt}}$ = k_i1_[$R^{•}$][A] + k_fa1_[$R^{•}$][$Z_{o}^{a}$] − k_aa1_[T][$Y_{o}^{a}$] − k_at3_[$Z_{o}^{b}$][$Y_{o}^{a}$] + k_ft3_[$Y_{o}^{b}$][${\;Z}_{o}^{a}$] + k_ft1_[$Y_{o}^{a}$][${\;Z}_{o}^{a}$] − k_at1_[$Z_{o}^{a}$][$Y_{o}^{a}$] − k_p11_[A][$Y_{o}^{a}$] − k_p12_[B][$Y_{o}^{a}$] + k_p11_[A][$Y_{o}^{a}$] + k_p21_[A][$Y_{o}^{b}$] − k_tc3_[$Y_{o}^{b}$][$Y_{o}^{a}$] − k_td3_[$Y_{o}^{b}$][${\;Y}_{o}^{a}$] − k_tc1_[$Y_{o}^{a}$][${\;Y}_{o}^{a}$] − k_td1_[$Y_{o}^{a}$][${\;Y}_{o}^{a}$]	(11)
$\frac{{\mathrm{dY}}_{o}^{b}}{\mathrm{dt}}$ = k_i2_[$R^{•}$][B] + k_fa2_[$R^{•}$][$Z_{o}^{b}$] − k_aa2_[T][$Y_{o}^{b}$] − k_at4_[$Z_{o}^{a}$][$Y_{o}^{b}$] + k_ft4_[$Y_{o}^{a}$][${\;Z}_{o}^{b}$] + k_ft2_[$Y_{o}^{b}$][${\;Z}_{o}^{b}$] − k_at2_[$Z_{o}^{b}$][$Y_{o}^{b}$] − k_p21_[A][$Y_{o}^{b}$] − k_p22_[B][$Y_{o}^{b}$] + k_p12_[B][$Y_{o}^{a}$] + k_p22_[B][${\;Y}_{o}^{b}$] − k_tc3_[$Y_{o}^{a}$][$Y_{o}^{b}$] − k_td3_[$Y_{o}^{a}$][$Y_{o}^{b}$] − k_tc2_[$Y_{o}^{b}$][$Y_{o}^{b}$] − k_td2_[$Y_{o}^{b}$][$Y_{o}^{b}$]	(12)
$\frac{{\mathrm{dZ}}_{o}^{a}}{\mathrm{dt}}$ = −k_fa1_[$R^{•}$][$Z_{o}^{a}$] + k_aa1_[T][$Y_{o}^{a}$] + k_at3_[$Z_{o}^{b}$][$Y_{o}^{a}$] − k_ft3_[$Y_{o}^{b}$][${\;Z}_{o}^{a}$] + k_at1_[$Z_{o}^{a}$][$Y_{o}^{a}$] − k_ft1_[$Y_{o}^{a}$][${\;Z}_{o}^{a}$]	(13)
$\frac{{\mathrm{dZ}}_{o}^{b}}{\mathrm{dt}}$ = −k_fa2_[$R^{•}$][$Z_{o}^{b}$] + k_aa2_[T][$Y_{o}^{b}$] − k_ft2_[$Y_{o}^{b}$][$Z_{o}^{b}$] + k_at2_[$Z_{o}^{b}$]$\;[Y_{o}^{b}$] + k_at4_[$Z_{o}^{a}$][$Y_{o}^{b}$] − k_ft4_[$Y_{o}^{a}$][$Z_{o}^{b}$]	(14)
$\frac{{\mathrm{dD}}_{o}}{\mathrm{dt}}$$=k{\mathrm{tc}}_{3}[Y_{o}^{a}$$][Y_{o}^{b}$$]+\frac{1}{2}$$k{\mathrm{tc}}_{1}[Y_{o}^{a}$$][Y_{o}^{a}$$]+\frac{1}{2}$$k{\mathrm{tc}}_{2}[Y_{o}^{b}$$][Y_{o}^{b}$$]+k{\mathrm{td}}_{3}[Y_{o}^{b}$$][Y_{o}^{a}$$]+k{\mathrm{td}}_{1}[Y_{o}^{a}$$][Y_{o}^{a}$$]+k{\mathrm{td}}_{2}[Y_{o}^{b}$$][{\;Y}_{o}^{b}$$]+k{\mathrm{td}}_{3}[Y_{o}^{b}$$][Y_{o}^{a}$]	(15)
First-order moments	
$\frac{{\mathrm{dY}}_{1}^{a}}{\mathrm{dt}}$ = k_i1_[$R^{•}$][A] + k_fa1_[$R^{•}$][$Z_{1}^{a}$] − k_aa1_[T][$Y_{1}^{a}$] − k_at3_[$Z_{o}^{b}$][$Y_{1}^{a}$] + k_ft3_[$Y_{o}^{b}$][$Z_{1}^{b}$] + k_ft1_[$Y_{o}^{a}$][$Z_{1}^{a}$] − k_at1_[$Z_{o}^{a}$]$\;[Y_{1}^{a}$] − k_p11_[A][$Y_{1}^{a}$] − k_p12_[B][$Y_{1}^{a}$] + k_p11_[A][${\;Y}_{1}^{a}$ + $Y_{o}^{a}$] + k_p21_[A][$Y_{1}^{b}$ + $Y_{o}^{b}$] − k_tc3_[$Y_{o}^{b}$][$Y_{1}^{a}$] − k_td3_[$Y_{o}^{b}$][$Y_{1}^{a}$] − k_tc1_[$Y_{o}^{a}$][$Y_{1}^{a}$] − k_td1_[$Y_{o}^{a}$][$Y_{1}^{a}$]	(16)
$\frac{{\mathrm{dY}}_{1}^{b}}{\mathrm{dt}}$ = k_i2_[$R^{•}$][B] + k_fa2_[$R^{•}$][$Z_{1}^{b}$] − k_aa2_[T][$Y_{1}^{b}$] − k_at4_[$Z_{o}^{a}$][$Y_{1}^{b}$] + k_ft4_[$Y_{o}^{a}$][$Z_{1}^{b}$] + k_ft2_[$Y_{o}^{b}$][$Z_{1}^{b}$] − k_at2_[$Z_{o}^{b}$][$Y_{1}^{b}$] − k_p21_[A][$Y_{1}^{b}$] − k_p22_[B][$Y_{1}^{b}$] + k_p12_[B][${\;Y}_{1}^{a}$ + $Y_{o}^{a}$] + k_p22_[B][$Y_{1}^{b}$ + $Y_{o}^{b}$] − k_tc3_[$Y_{o}^{a}$][$Y_{1}^{b}$] − k_td3_[$Y_{o}^{a}$][$Y_{1}^{b}$] − k_tc2_[$Y_{o}^{b}$][$Y_{1}^{b}$] − k_td2_[$Y_{o}^{b}$][$Y_{1}^{b}$]	(17)
$\frac{{\mathrm{dZ}}_{1}^{a}}{\mathrm{dt}}$ = −k_fa1_[$R^{•}$][$Z_{1}^{a}$] + k_aa1_[T][$Y_{1}^{a}$] + k_at3_[$Z_{o}^{b}$][$Y_{1}^{a}$] − k_ft3_[$Y_{o}^{b}$][$Z_{1}^{a}$] + k_at1_[$Z_{o}^{a}$][$Y_{1}^{a}$] − k_ft1_[$Y_{o}^{a}$][$Z_{1}^{a}$]	(18)
$\frac{{\mathrm{dZ}}_{1}^{b}}{\mathrm{dt}}$ = −k_fa2_[$R^{•}$][$Z_{1}^{b}$] + k_aa2_[T][$Y_{1}^{b}$] − k_ft2_[$Y_{o}^{b}$][$Z_{1}^{b}$] + k_at2_[$Z_{o}^{b}$][$Y_{1}^{b}$] + k_at4_[$Z_{o}^{a}$][$Y_{1}^{b}$]− k_ft4_[$Y_{o}^{a}$][$Z_{1}^{b}$]	(19)
$\frac{{\mathrm{dD}}_{1}}{\mathrm{dt}}$$=k{\mathrm{tc}}_{3}[Y_{o}^{a}Y_{1}^{b}$$+Y_{1}^{a}Y_{o}^{b}$$]+\frac{1}{2}$$k{\mathrm{tc}}_{1}[Y_{o}^{a}Y_{1}^{a}$$+Y_{1}^{a}Y_{o}^{a}$$]+\frac{1}{2}$$k{\mathrm{tc}}_{2}[Y_{o}^{b}Y_{1}^{b}$$+Y_{1}^{b}Y_{o}^{b}$$]+k{\mathrm{td}}_{3}[Y_{o}^{b}$$][Y_{1}^{a}$$]+k{\mathrm{td}}_{1}[Y_{o}^{a}$$][Y_{1}^{a}$$]+k{\mathrm{td}}_{2}[Y_{o}^{b}$$][Y_{1}^{b}$$]+k{\mathrm{td}}_{3}[Y_{o}^{a}$$][Y_{1}^{b}$]	(20)
Second-order moments	
$\frac{{\mathrm{dY}}_{2}^{a}}{\mathrm{dt}}$ = k_i1_[$R^{•}$][A] + k_fa1_[$R^{•}$][$Z_{2}^{a}$] − k_aa1_[T][$Y_{2}^{a}$] − k_at3_[$Z_{o}^{b}$][$Y_{2}^{a}$] + k_ft3_[$Y_{o}^{b}$][$Z_{2}^{a}$] + k_ft1_[$Y_{o}^{a}$][$Z_{2}^{a}$] − k_at1_[$Z_{o}^{a}$][$Y_{2}^{a}$] − k_p11_[A][$Y_{2}^{a}$] − k_p12_[B][${\;Y}_{2}^{a}$] + k_p11_[A][${\;Y}_{2}^{a}$ + 2$Y_{1}^{a}$ + $Y_{o}^{a}$] + k_p21_[A][${\;Y}_{2}^{b}$ + 2$Y_{1}^{b}$ + $Y_{o}^{b}$] − k_tc3_[$Y_{o}^{b}$][$Y_{2}^{a}$] − k_td3_[$Y_{o}^{b}$][$Y_{2}^{a}$] − k_tc1_[$Y_{o}^{a}$][$Y_{2}^{a}$] − k_td1_[$Y_{o}^{a}$][$Y_{2}^{a}$]	(21)
$\frac{{\mathrm{dY}}_{2}^{a}}{\mathrm{dt}}$ = k_i2_[$R^{•}$][B] + k_fa2_[$R^{•}$][$Z_{2}^{b}$] − k_aa2_[T][$Y_{2}^{b}$] − k_at4_[$Z_{o}^{a}$][$Y_{2}^{b}$] + k_ft4_[$Y_{o}^{a}$][$Z_{2}^{b}$] + k_ft2_[$Y_{o}^{b}$][$Z_{2}^{b}$] − k_at2_[$Z_{o}^{b}$][$Y_{2}^{b}$] − k_p21_[A][$Y_{2}^{b}$] − k_p22_[B][$Y_{2}^{b}$] + k_p12_[B][${\;Y}_{2}^{a}$ + 2$Y_{1}^{a}$ + $Y_{o}^{a}$] + k_p22_[B][${\;Y}_{2}^{b}$ + 2$Y_{1}^{b}$ + $Y_{o}^{b}$] − k_tc3_[$Y_{o}^{a}$][$Y_{2}^{b}$] − k_td3_[$Y_{o}^{a}$][$Y_{2}^{b}$] − k_tc2_[$Y_{o}^{b}$][$Y_{2}^{b}$] − k_td2_[$Y_{o}^{b}$][$Y_{2}^{b}$]	(22)
$\frac{{\mathrm{dZ}}_{2}^{a}}{\mathrm{dt}}$ = −k_fa1_[$R^{•}$][$Z_{2}^{a}$] + k_aa1_[T][$Y_{2}^{a}$] + k_at3_[$Z_{o}^{b}$][$Y_{2}^{a}$] − k_ft3_[$Y_{o}^{b}$][$Z_{2}^{a}$] + k_at1_[$Z_{o}^{a}$][$Y_{2}^{a}$] − k_ft1_[$Y_{o}^{a}$][$Z_{2}^{a}]$	(23)
$\frac{{\mathrm{dZ}}_{2}^{b}}{\mathrm{dt}}$ = −k_fa2_[$R^{•}$][$Z_{2}^{b}$] + k_aa2_[T][$Y_{2}^{b}$] − k_ft2_[$Y_{o}^{b}$][$Z_{2}^{b}$] + k_at2_[$Z_{o}^{b}$][$Y_{2}^{b}$] + k_at4_[$Z_{o}^{a}$][$Y_{2}^{b}$] − k_ft4_[$Y_{o}^{a}$][$Z_{2}^{b}$]	(24)
$\frac{{\mathrm{dD}}_{2}}{\mathrm{dt}}$$=k{\mathrm{tc}}_{3}[Y_{o}^{a}Y_{2}^{b}$$+2Y_{1}^{a}Y_{1}^{b}$$+Y_{2}^{a}Y_{o}^{b}$$]+\frac{1}{2}$$k{\mathrm{tc}}_{1}[Y_{o}^{a}Y_{2}^{a}$$+2Y_{1}^{a}Y_{1}^{a}$$+Y_{2}^{a}Y_{o}^{a}$$]+\frac{1}{2}$$k{\mathrm{tc}}_{2}[Y_{o}^{b}Y_{2}^{b}$$+2Y_{1}^{b}Y_{1}^{b}$$+Y_{2}^{b}Y_{o}^{b}$$]+k{\mathrm{td}}_{3}[Y_{o}^{b}$$][Y_{2}^{a}$$]+k{\mathrm{td}}_{1}[Y_{o}^{a}$$][Y_{2}^{a}$$]+k{\mathrm{td}}_{2}[Y_{o}^{b}$$][Y_{2}^{b}$$]+k{\mathrm{td}}_{3}[Y_{o}^{a}$$][Y_{2}^{b}$]	(25)

**Table 5:** Kinetic constants for RAFT copolymerization of St (1)-GMA (2).

PM_1_ = 104.15 gmol^−1^	
PM_2_ = 142.20 gmol^−1^	
k_d_ (s^−1^) = 2.2798 × 10^19^ exp(−166.9 kJmol^−1^/RT) initiator	[28]
f = 0.60	
k_p11_ (L mol^−1^ s^−1^) = 4.266 × 10^7^ exp(−3909.61/T) St	[29]
k_p22_ (L mol^−1^ s^−1^) = 5.100 × 10^6^ exp(−2754/T) GMA	[30]
k_i1_ (L mol^−1^ s^−1^) = k_p11_	
k_i2_ (L mol^−1^ s^−1^) = k_p22_	
r_1_ (k_p11_/k_p12_) = 0.3724	[19]
r_2_ (k_p22_/k_p21_) = 0.6834	[19]
k_tc1_ (L mol^−1^ s^−1^) = 2.0 × 10^10^ exp(−1553.01/T) St	[31]
k_tc2_ (L mol^−1^ s^−1^) = 2.57 × 10^8^ exp(−292.0/T) ^(a)^ GMA	[32]
$k{\mathrm{tc}}_{3}\;(L\;{\mathrm{mol}}^{-1}\;s^{-1})=\sqrt{k_{\mathrm{tc}1}k_{\mathrm{tc}2}}$	
k_td_ = k_tc_	

^(a)^ Assumed equal to the corresponding value for butyl acrylate (see [32]).

**Table 6:** Physical properties of monomers and polymer for calculation of fractional free volume.

$\rho_{M_{1}}$ (g mL^−1^) = 0.9193 − 0.000665 T/°C	[35]
$\rho_{M_{2}}$ (g mL^−1^) = 1.09428 − 0.001041 T/°C	[36]
$\rho_{P_{1}}$ (g mL^−1^) = 1.09926 − 2.65 × 10^−4^ T/°C	[35]
$\rho_{P_{2}}$ (g mL^−1^) = 1.08	This work
$T_{g,M_{1}}$ (K) = 156.15	[37]
$T_{g,M_{2}}$ (K) = 348.15	[38]
$T_{g,P_{1}}$ (K) = 373.15	[39]
$T_{g,P_{2}}$ (K) = 347.0	[40]
$\alpha_{M_{1}}$ (K^−1^) = 0.00062	[41]
$\alpha_{M_{2}}$ (K^−1^) = 0.0004	This work
$\alpha_{P_{1}}$ (K^−1^) = 0.00045	[39]
$\alpha_{P_{2}}$ (K^−1^) = 0.00048 ^(a)^	[41]
$\alpha_{p}$ (K^−1^) = F_1_ $\alpha_{P_{1}}$ + F_2_ $\alpha_{P_{2}}$	[42]

^(a)^ Assumed equal to the corresponding value for butyl acrylate (see [36]).

**Table 7:** Simplifications made about the RAFT activation and transfer kinetic rate constants.

St homopolymerization				
RAFT activation	k_aa1_ = k_fa1_	RAFT transfer	k_at1_ = k_ft1_	k_aa1_ = k_at1_
GMA homopolymerization				
RAFT activation	k_aa2_ = k_fa2_	RAFT transfer	k_at2_ = k_ft2_	k_aa2_ = k_at2_

**Table 8:** Summary of experimental conditions used in this study; T = 103 °C; [St + GMA]_o_:[CPDT]_o_:[ACHN]_o_ = R_3_:R_2_:R_1_.

Copolymer Identifier	Styrene [St]_o_	GMA [GMA]_o_	RAFT [CPDT]_o_	Initiator [ACHN]_o_	R_3_:R_2_:R_1_
	(mol L^−1^)	(mol L^−1^)	(mol L^−1^)	(mol L^−1^)	
St-GMA 100-00	8.6445	–	0.0297	0.0074	291:4:1
St-GMA 90-10	7.6091	0.8455	0.0304	0.0076	278:4:1
St-GMA 85-15	7.1370	1.2595	0.0307	0.0077	273:4:1
St-GMA 70-30	5.7546	2.4662	0.0316	0.0079	260:4:1
St-GMA 60-40	4.8669	3.2446	0.0322	0.0080	251:4:1
St-GMA 00-100	–	7.5949	0.0360	0.0090	211:4:1

**Table 9:** Activation and transfer kinetic rate constants evaluated in this study.

RAFT activation, L mol^−1^ s^−1^	
k_fa1_ = 101,113 ± 2690	k_aa1_ = 101,113 ± 2690
k_fa2_ = 484,000 ± 1200	k_aa2_ = 484,000 ± 1200
RAFT transfer, L mol^−1^ s^−1^	
k_ft1_ = 101,113 ± 2690	k_at1_ = 101,113 ± 2690
k_ft2_ = 484,000 ± 1200	k_at2_ = 484,000 ± 1200
k_ft3_ = 271,517 ± 7223	k_at3_ = 708,224 ± 1756
k_ft4_ = 708,224 ± 1756	k_at4_ = 271,517 ± 7223

**Table 10:** AA termination parameters determined in this study.

Sample	St-GMA					
	100-00	90-10	85-15	70-30	60-40	00-100
βk_tc_ = βk_td_	0.3836 ± 0.0023	0.3581 ± 0.0214	0.3979 ± 0.0001	0.23045 ± 5 × 10^−5^	0.2925 ± 5 × 10^−^^5^	0.00081 ± 7 × 10^−7^

## Data Availability

Data is contained within the article.
